# 5-nm LiF as an Efficient Cathode Buffer Layer in Polymer Solar Cells Through Simply Introducing a C_60_ Interlayer

**DOI:** 10.1186/s11671-017-2299-y

**Published:** 2017-09-21

**Authors:** Xiaodong Liu, L. Jay Guo, Yonghao Zheng

**Affiliations:** 1 School of Optoelectronic Information, University of Electronic Science and Technology of China (UESTC), Chengdu, 610054 People’s Republic of China; 2 Center for Applied Chemistry, University of Electronic Science and Technology of China (UESTC), Chengdu, 610054 People’s Republic of China; 3 Department of Electrical Engineering and Computer Science, The University of Michigan, 1301 Beal Ave., Ann Arbor, MI 48109 USA

**Keywords:** Polymer solar cells, Thick LiF buffer layer, C_60_/LiF bilayer, Mixed morphology

## Abstract

Lithium fluoride (LiF) is an efficient and widely used cathode buffer layer (CBL) in bulk heterojunction polymer solar cells (PSCs). The LiF thickness is normally limited to 1 nm due to its insulting property. Such small thickness is difficult to precise control during thermal deposition, and more importantly, 1-nm-thick LiF cannot provide sufficient protection for the underlying active layer. Herein, we demonstrated the application of a very thick LiF as CBL without sacrificing the device efficiency by simply inserting a C_60_ layer between the active layer and LiF layer. The devices with the C_60_/LiF (5 nm) double CBLs exhibit a peak power conversion efficiency (PCE) of 3.65%, which is twofold higher than that (1.79%) of LiF (5 nm)-only device. The superior performance of the C_60_/LiF (5 nm)-based devices is mainly attributed to the good electrical conductivity of the C_60_/LiF (5 nm) bilayer, arising from the intermixing occurred at the C_60_/LiF interface. Besides, the formation of a P3HT/C_60_ subcell and the optical spacer effect of C_60_ also contribute to the increase in short-circuit current density (*J*_sc_) of the device. With further increase of LiF thickness to 8 nm, a PCE of 1.10% is attained for the C_60_/LiF-based device, while the negligible photovoltaic performance is observed for the LiF-only device. All in all, our results show that C_60_/LiF bilayer is a promising alternative to LiF single layer due to its high tolerance to the LiF thickness variations.

**Electronic supplementary material:**

The online version of this article (10.1186/s11671-017-2299-y) contains supplementary material, which is available to authorized users.

## Background

Solution-processed bulk heterojunction polymer solar cells (PSCs) have received increasing attention in recent decades because of their potential advantages such as low cost, light weight, and possibility to fabricate large-scale, flexible, and semitransparent devices [1–5]. By far, the relatively low power conversion efficiency (PCE) compared to silicon-based solar cells is still a major limitation that hinders their practical application. To achieve commercialization of this promising technology, extensive research efforts have focused on increasing the efficiency of PSCs. Until now, PCEs in the range of 11–13% have been demonstrated, primarily owing to the development of novel conjugated polymer donor and non-fullerene acceptor materials [6–12]. Besides, the introduction of anode/cathode buffer layer between the active layer and the electrode provides another efficient means to improve the device performance [13–21].

PSCs can be divided into conventional and inverted structures according to whether the indium-tin-oxide (ITO) electrode serves as the anode or the cathode. For the conventional PSCs with ITO as anode, a low work function metal such as Ca is commonly used as cathode buffer layer (CBL) to reduce the work function of the cathode (e.g., Al, Ag). However, Ca is easily oxidized when exposed to air, resulting in the poor stability of the devices. Another widely used CBL in PSCs is lithium fluoride (LiF), which has been demonstrated to enhance the device performance through the formation of an interfacial dipole at the cathode interface [22]. Nevertheless, the thickness of LiF is limited to less than 2 nm (generally ~ 1 nm) due to its insulating property [23, 24]. Such a small thickness is very difficult to be controlled via thermal deposition. Furthermore, 1-nm-thick LiF cannot provide sufficient protection for the underlying active layer during the evaporation of hot metal atoms [17, 25].

To address these problems, we have previously reported five stacks of C_60_/LiF CBL, which substantially improved the device efficiency and stability of PSCs due to its good electrical conductivity even though a very thick LiF was used [26]. However, the five-stacked C_60_/LiF film was prepared by alternating deposition of C_60_ and LiF layers. This preparation process is very complicated and time-consumed, and significantly increases the cost of device fabrication. In this work, we adopted a C_60_/LiF bilayer as CBL to achieve the same effect as five-stacked C_60_/LiF CBL. After depositing a C_60_ layer prior to the LiF evaporation, a thick LiF is allowed to be used without sacrificing the device efficiency. The PSCs with C_60_/LiF double CBLs maintained a ~ 3% PCE over a wide range of LiF thickness (1~6 nm), and showed a PCE of 1.10% even at a very thick LiF, 8 nm. In contrast, the PSCs with LiF single CBL exhibited a rapid decrease of PCE with increasing LiF thickness and had negligible photovoltaic performance at LiF thickness of 8 nm. Besides, the peak efficiency (3.77%) of C_60_/LiF-based devices is ~ 23% higher than that (3.06%) of LiF-only device. Taken all together, these results indicate that C_60_/LiF bilayer is a more promising candidate as a CBL compared to single LiF layer.

## Methods

### Fabrication of PSCs

ITO-coated glass substrates (Delta Technologies, LTD) were cleaned in acetone and isopropyl alcohol (IPA) under sonication for 5 min each and then treated by O_2_ plasma for 60 s to generate the hydrophilic surface. The filtered poly(3,4-ethylenedioxythiophene):poly(styrenesulfonate) (PEDOT:PSS) solution (H. C. Starck, Clevios PH 500) was spin-coated onto the cleaned glass/ITO substrates at a speed of 2000 rpm for 50 s, followed by baking at 110 °C for 20 min under nitrogen atmosphere. Subsequently, the samples were transferred to a N_2_-purged glovebox (< 0.1 ppm O_2_ and H_2_O) for spin-coating of photoactive layer.

P3HT (Rieke Metals Inc., 4002-EE, 91–94% regioregularity) and PCBM (American Dye Source, purity > 99.5%) were dissolved in chlorobenzene with a weight ratio of 1:1. The mixed solution was filtered using a 0.45 μm filter and then spin-coated on top of the PEDOT:PSS layer at 1000 rpm for 50 s, followed by thermal annealing at 130 °C for 20 min, which produced a ~ 160-nm-thick active layer measured using a Dektek surface profiler. The C_60_, LiF, and Al (75 nm) electrode were sequentially deposited by thermal evaporation at a base pressure of 1 × 10^− 6^ mbar. The deposition rate and film thickness were monitored with a quartz crystal sensor. A circular-shaped shadow mask of 1 mm diameter was put on the sample to define the active area before the Al deposition.

### Characterization

The current density-voltage (*J*-*V*) characteristics were measured using a Keithley 2400 system under simulated Air Mass 1.5 Global (AM 1.5 G) solar illumination at an intensity of 100 mW/cm^2^, which was calibrated by a power meter (OPHIR, Nova-Oriel) and a reference silicon solar cell. The measurements were carried out with the PSCs inside the glovebox. Atomic force microscope (AFM) images were taken with a Veeco Dimension-Icon AFM operated in tapping mode. Absorption spectra were obtained using a Varian Cary 50 UV/Vis spectrophotometer. Photo-induced charge extraction by linearly increasing voltage (Photo-CELIV) measurements were performed on PSCs under ambient conditions. A pulsed N_2_ laser (337.1 nm, 1.4 ns) was used to generate the charge carriers, which were then extracted by a reverse-bias voltage ramp that was applied after 100 μs delay time. The current transients were recorded using a digital storage oscilloscope (50 Ω input impedance). During and after illumination, an offset voltage was applied to compensate the built-in potential of the devices, which prevents an initial photocurrent prior to the application of the voltage ramp. The mobility of the carriers can be calculated according to the following equation [27, 28]:

1 $$ \mu =\frac{2{d}^2}{3{At}_{\mathrm{max}}^2\left[1+0.36\frac{\varDelta j}{j(0)}\right]} $$

where *μ* is the charge carrier mobility, *d* is the thickness of the active layer, *A* is the voltage rise speed, *t*_max_ is the time when the extraction current reaches the maximum value, ∆*j* is the current extraction peak height, and *j*(0) is the displacement current of the capacitance.

## Results and Discussion

**Fig. 1:**
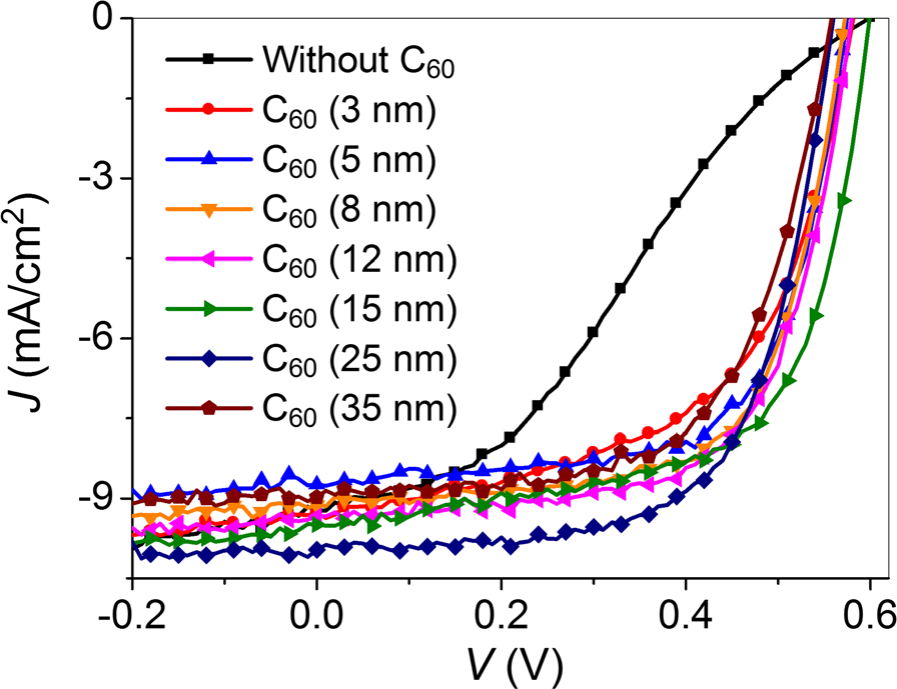
*J*-*V* characteristics, recorded under 100 mW/cm^2^ illumination (AM 1.5 G), of the PSCs with and without different thicknesses of C_60_ inserted between P3HT:PCBM and 5-nm-thick LiF layer

Figure 1 shows the *J*-*V* characteristics, recorded under 100 mW/cm^2^ illumination (AM 1.5 G), of the PSCs with and without different thicknesses of C_60_ sandwiched between the active layer and 5-nm-thick LiF layer. The device without the C_60_ layer shows S-shaped curve, resulting in the low fill factor (FF) and therefore the low PCE, despite the typical short-circuit current density (*J*_sc_) and open-circuit voltage (*V*_oc_). The low FF is rationalized in terms of the insulating property of LiF, which blocks the electron injection/extraction when the LiF layer is too thick and thus leads to the large series resistance (*R*_s_) and small shunt resistance (*R*_sh_) of the device as shown in Table 1 (*R*_s_ and *R*_sh_ were calculated from the inverse slope of photo *J*-*V* curve at 0 mA/cm^2^ and 0 V, respectively). As for the *J*_sc_, the normal value (9.23 mA/cm^2^) implies that the built-in electric filed inside the device (from work function difference between anode and cathode) is sufficient to promote the electron transport through LiF (5 nm) CBL by tunneling. After introducing 3-nm-thick C_60_ layer between P3HT:PCBM and LiF (5 nm) layers, the S-shape disappears and the FF increases significantly from 32.4 to 56.3%. The increased FF arises from the reduced *R*_s_, which implies that the C_60_ (3 nm)/LiF (5 nm) bilayer possesses better electrical conductivity than single LiF (5 nm) layer. With the increase of C_60_ thickness, the FF first increases, reaching a maximum value of 67% at 8 nm and then decreases slightly with further increasing C_60_ thickness. Due to the recovery of FF, the C_60_/LiF (5 nm)-based devices show a maximum PCE of 3.65%, which is two times higher than that (1.79%) of LiF (5 nm)-only device. To demonstrate the reproducibility of the results, the average photovoltaic parameters and standard deviations of the studied devices were calculated from a batch of five devices, as shown in Additional file 1: Table S1. For each device, all the parameters including *J*_sc_, *V*_oc_, FF, and PCE are highly reproducible with little variation, which validates the reliability of the results presented in Table 1.

**Table 1:** Photovoltaic parameters for the P3HT:PCBM-based PSCs with and without different thicknesses of C_60_ inserted between the active layer and 5-nm-thick LiF layer

CBL	*J*_sc_ (mA/cm^2^)	*V*_oc_ (V)	FF (%)	PCE (%)	*R*_s_ (Ω cm^2^)	*R*_sh_ (Ω cm^2^)
LiF	9.23	0.60	32.4	1.79	87.21	232.70
C_60_ (3 nm)/LiF	9.28	0.58	56.3	3.03	7.74	293.46
C_60_ (5 nm)/LiF	8.74	0.57	66.7	3.32	6.03	732.93
C_60_ (8 nm)/LiF	9.13	0.57	67.0	3.48	6.22	768.36
C_60_ (12 nm)/LiF	9.34	0.58	65.1	3.53	6.14	655.67
C_60_ (15 nm)/LiF	9.49	0.60	64.0	3.65	5.24	351.63
C_60_ (25 nm)/LiF	9.97	0.56	65.3	3.65	6.13	726.52
C_60_ (35 nm)/LiF	8.97	0.56	61.9	3.11	8.19	751.58

**Fig. 2:**
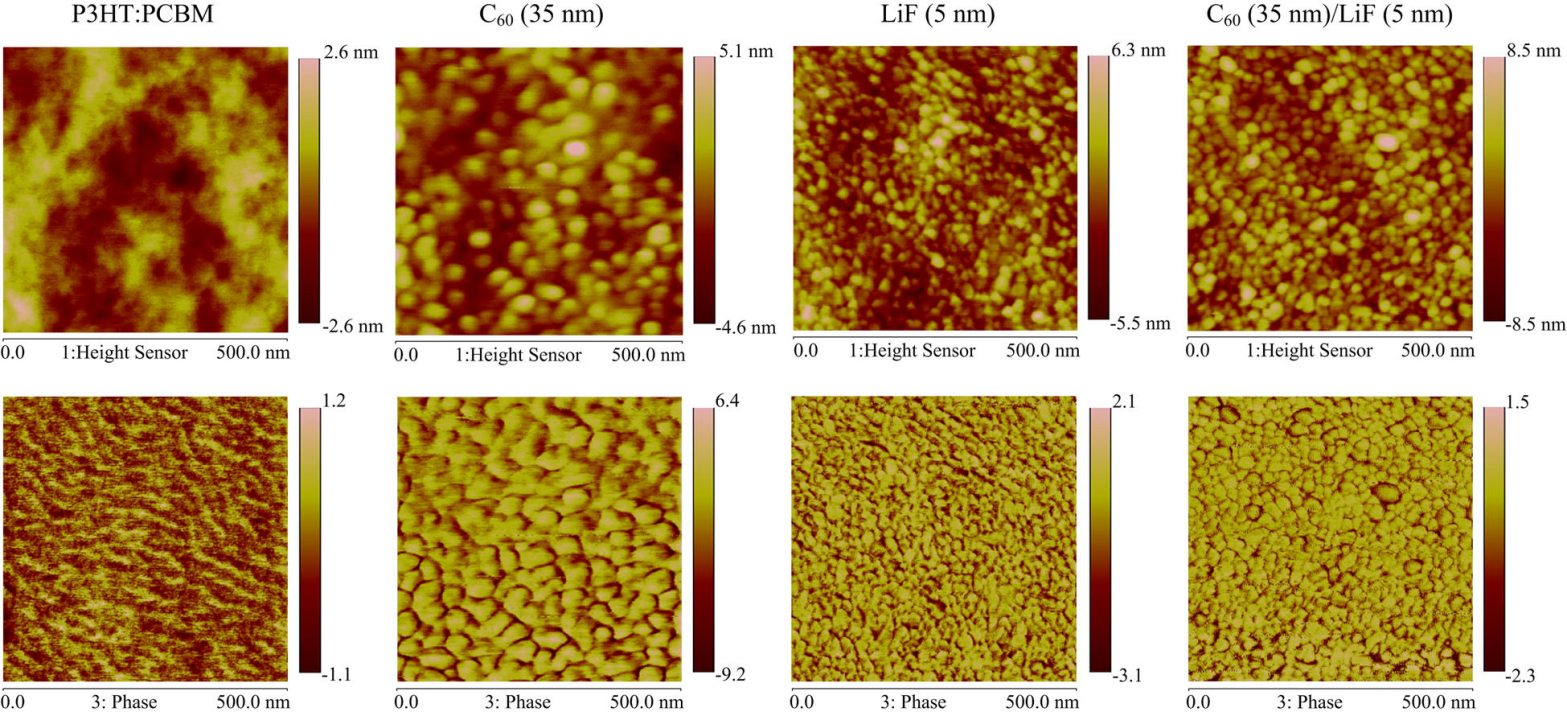
Tapping mode AFM height (top) and phase (bottom) images of P3HT:PCBM, P3HT:PCBM/C_60_ (35 nm), P3HT:PCBM/LiF (5 nm), and P3HT:PCBM/C_60_ (35 nm)/LiF (5 nm) films. The corresponding root-mean-square (rms) roughnesses are 0.81, 1.36, 1.67, and 2.18 nm, respectively

To find out the reasons leading to the high FF for the C_60_/LiF (5 nm)-based PSCs, AFM measurements were performed to examine the morphology of the LiF layer on the C_60_ surface. Figure 2 shows the height (top) and phase (bottom) images, recorded by tapping mode AFM, of the P3HT:PCBM films without and with the C_60_ (35 nm), LiF (5 nm), and C_60_ (35 nm)/LiF (5 nm) layers deposited on top (image size 500 nm × 500 nm). The pristine P3HT:PCBM film exhibits a very smooth surface with a low root-mean-square (rms) roughness of 0.81 nm (height image) and shows fibrillar crystalline domains of P3HT (phase image) [29]. After depositing 35-nm-thick C_60_ and 5-nm-thick LiF on top, the rms roughness increases to 1.36 and 1.67 nm, respectively. Although there is no significant difference in rms roughness between the top C_60_ and LiF layers, the surface morphologies of these two films are very different. The 35-nm-thick C_60_ shows larger aggregates (spherical shape) as compared to 5-nm-thick LiF, which can also be observed in their phase images. When depositing the C_60_ (35 nm)/LiF (5 nm) bilayer on the P3HT:PCBM film, both the C_60_ (large size) and LiF (small size) aggregates are observed, indicating that the underlying C_60_ layer is not completely covered by 5-nm-thick LiF. Therefore, some intermixing occurs at the C_60_/LiF interface, which results in the good electrical conductivity of C_60_/LiF (5 nm) bilayer considering the percolation path formed by C_60_ molecules.

**Fig. 3:**
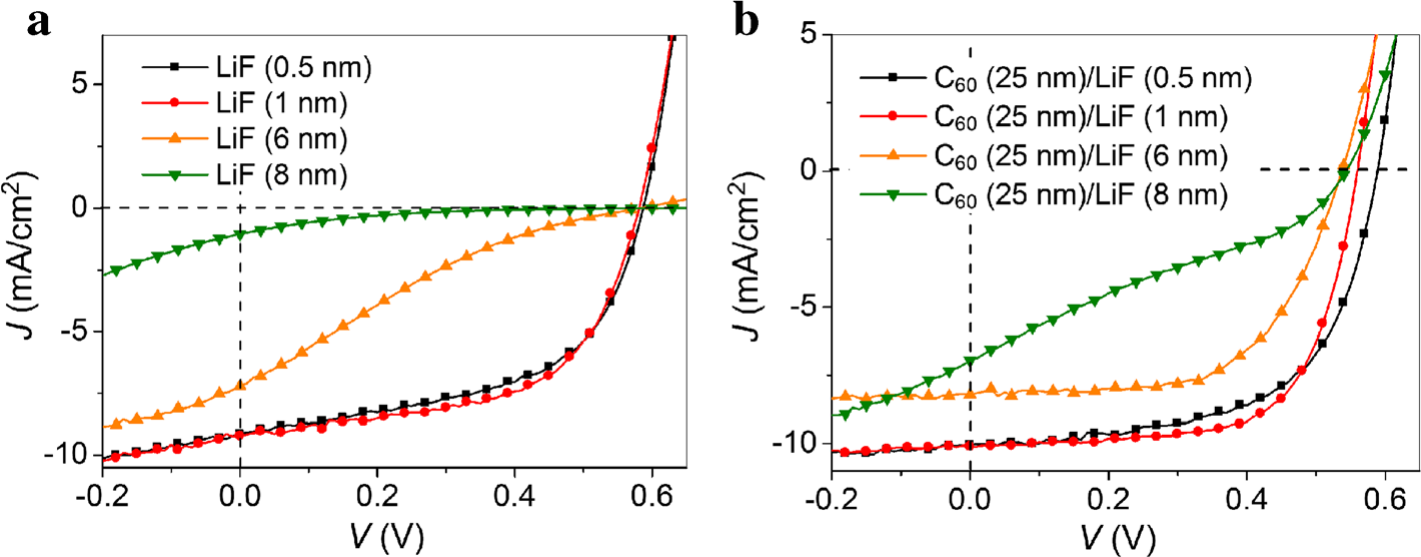
*J*-*V* characteristics, recorded under 100 mW/cm^2^ illumination (AM 1.5 G), of the PSCs using **a** LiF single and **b** C_60_ (25 nm)/LiF double CBLs with different thicknesses of LiF

To further investigate the influence of the C_60_/LiF double CBLs on the device performance of PSCs, we fix the C_60_ thickness at the optimum value of 25 nm while changing the LiF thickness from 0.5 to 8 nm. For comparison, the devices with LiF single CBL were also fabricated. Figure 3 shows the *J*-*V* characteristics, recorded under 100 mW/cm^2^ illumination (AM 1.5 G), of the PSCs using LiF single and C_60_/LiF double CBLs with varying thicknesses of LiF. The corresponding photovoltaic parameters of the devices are summarized in Table 2. The devices with LiF single CBL have a maximum PCE of 3.06% at the optimal LiF thickness of 1 nm. Further increasing the thickness leads to a rapid decrease in PCE to 0.79% at 6 nm and 0.06% at 8 nm. In contrast, the devices with C_60_ (25 nm)/LiF double CBLs exhibit improved performance with a peak efficiency of 3.77% at the LiF thickness of 1 nm. More importantly, as the thickness increases to 6 and 8 nm, PCEs of 2.65 and 1.10% are attained, respectively, which are significantly higher than those of LiF-only devices. It should be mentioned that the results presented in Table 2 is also highly reproducible, as demonstrated by the very small standard deviations of the device characteristic parameters (Additional file 1: Table S2). For instance, the standard deviation of the device efficiency is less than 0.2% (0.1% for most devices), indicating high reproducibility. Furthermore, the average PCE shows the same trend as observed in Table 2, which implies that the comparison of efficiency among different groups is reliable.

**Table 2:** Photovoltaic parameters for the P3HT:PCBM-based PSCs using LiF single and C_60_ (25 nm)/LiF double CBLs with different thicknesses of LiF

CBL	*J*_sc_ (mA/cm^2^)	*V*_oc_ (V)	FF (%)	PCE (%)	*R*_s_ (Ω cm^2^)	*R*_sh_ (Ω cm^2^)
LiF (0.5 nm)	9.15	0.59	53.8	2.91	5.50	208.94
LiF (1 nm)	9.21	0.58	57.2	3.06	6.14	253.56
LiF (6 nm)	7.20	0.58	18.9	0.79	195.21	62.65
LiF (8 nm)	1.05	0.58	10.2	0.06	4749.92	175.15
C_60_/LiF (0.5 nm)	10.03	0.59	60.2	3.57	4.95	323.33
C_60_/LiF (1 nm)	10.10	0.56	66.6	3.77	5.05	626.63
C_60_/LiF (6 nm)	8.21	0.54	59.8	2.65	8.98	744.54
C_60_/LiF (8 nm)	6.96	0.54	29.2	1.10	14.08	82.15

As shown in Table 2, the improvement in PCE for the C_60_ (25 nm)/LiF-based PSCs mainly arises from the increase in FF and *J*_sc_ due to the reduced *R*_s_. To better understand the *R*_s_ reduction, we investigate the charge transport properties of the LiF single layer and C_60_/LiF bilayer using the photo-CELIV technique [30, 31]. Additional file 1: Figure S1 shows the photo-CELIV current transients, recorded at varying voltage rise speeds, for the PSCs with the LiF single and C_60_/LiF double CBLs. In photo-CELIV, the time of extraction current maximum (*t*_max_) is used for estimating the charge carrier mobility according to Eq. 1 [27]. The calculated mobilities of the LiF (6 nm)-only device are 3.71, 3.40, and 3.59 × 10^− 5^ cm^2^ V^− 1^ s^− 1^ for the voltage slopes of 10, 20, and 30 kV/s, respectively, implying that the mobility is independent on the voltage rise speed. In contrast, the estimated mobilities of the C_60_ (25 nm)/LiF (6 nm)-based device are 3.81, 3.56, and 3.09 × 10^− 4^ cm^2^ V^− 1^ s^− 1^ for the voltage slopes of 10, 20, and 30 kV/s, respectively, which are one order of magnitude higher than those of the LiF (6 nm)-only device. The increased mobility after introducing a C_60_ layer can be attributed to the improved electrical conductivity arising from the intermixing occurred at the C_60_/LiF interface. In addition, it is noted that the photo-CELIV peak for the LiF (6 nm)-only device is broader than that for the C_60_ (25 nm)/LiF (6 nm)-based device, which indicates a more dispersive charge transport resulting from the larger imbalance between the electron and hole mobilities [32, 33]. This imbalance is attributed to the extremely low electron mobility for the LiF (6 nm)-only device considering that the extraction of electrons is blocked by the thick LiF layer. The accumulated electrons at the P3HT:PCBM/LiF interface screen the applied electric field and thereby decrease the rate of charge extraction in the device. In contrast, the narrow peak for the C_60_ (25 nm)/LiF (6 nm)-based device implies the balanced electron and hole mobilities as well as the improved electron extraction owing to the good conductivity of the C_60_ (25 nm)/LiF (6 nm) bilayer.

**Fig. 4:**
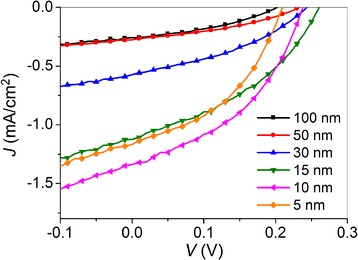
*J*-*V* characteristics of the PSCs with the device structure of ITO/PEDOT:PSS/P3HT (*x* nm)/C_60_ (25 nm)/LiF (1 nm)/Al using varying thicknesses of P3HT

Besides the significant improvement in FF, the *J*_sc_ is slightly enhanced after incorporation of C_60_ (25 nm) layer. Considering that the spin-coated P3HT:PCBM blend film consists of a P3HT-rich region near the top surface [34, 35], we speculate that the excitons generated in this region can be dissociated at the P3HT/C_60_ interface for C_60_ (25 nm)/LiF-based devices, which leads to the increased *J*_sc_ compared to the devices without the C_60_ interlayer. To verify this speculation, we fabricated the PSCs with a device structure of ITO/PEDOT:PSS/P3HT/C_60_ (25 nm)/LiF/Al, where the thickness of P3HT is varied from 5 to 100 nm. Figure 4 shows the *J-V* characteristics of these devices under 100 mW/cm^2^ illumination (AM 1.5 G), and the corresponding photovoltaic parameters are summarized in Additional file 1: Table S3. It is found that the *J*_sc_ of P3HT/C_60_-based solar cells increases as the P3HT thickness decreases, which is rationalized in terms of the limited exciton diffusion length in P3HT (~ 10 nm). The *J*_sc_ reaches a maximum value of 1.34 mA/cm^2^ at the P3HT thickness of 10 nm and then drops with further decease of the thickness to 5 nm due to the insufficient absorption. As mentioned above, such P3HT/C_60_ subcell is most likely formed after depositing 25-nm-thick C_60_ on top of the P3HT:PCBM active layer, which results in a 1.34 mA/cm^2^ increase in *J*_sc_ under ideal conditions for C_60_/LiF-based devices [36]. By comparing the *J*_sc_ values of the devices with and without the C_60_ (25 nm) interlayer, the enhancement in *J*_sc_ is around 1 mA/cm^2^ (except for the LiF (8 nm)-based devices), which is consistent with our speculation.

After introducing a C_60_ layer between the P3HT:PCBM and LiF layers, the optical field distribution within the solar cell is most likely altered, which will cause the variation in *J*_sc_ [26, 37]. To investigate this effect, we first simulated the electric field intensity inside the P3HT:PCBM active layer for the devices with and without the C_60_ interlayer. As shown in Additional file 1: Figure S2a, the integrated field intensity for the devices incorporating a C_60_ layer is weaker in the short-wavelength region and stronger in the long-wavelength region as compared to the device without C_60_ interlayer. This trend becomes more remarkable, and simultaneously, a red shift is observed with increasing the C_60_ thickness. Additional file 1: Figure S2b shows the absorption spectra of the pristine C_60_ film, and the P3HT:PCBM films with and without different CBLs deposited on top. Comparing the absorption spectra of P3HT:PCBM/C_60_ (25 nm) films with and without 8-nm-thick LiF, the two curves overlap almost completely, indicating that LiF does not absorb visible light. On the other hand, the P3HT:PCBM/C_60_ films have higher absorption in the wavelength ranges of 400~510 nm and 580~680 nm when compared to the pristine P3HT:PCBM film. This absorption enhancement becomes more pronounced with increasing C_60_ thickness. Intuitively, the absorption enhancement in the 400~510 nm wavelength range arises from the C_60_ absorption (400~550 nm). Additional file 1: Figure S2c shows the incident photon-to-current conversion efficiency (IPCE) spectra of the PSCs with LiF (5 nm) single and C_60_ (25 nm)/LiF (5 nm) double CBLs. Compared to the LiF-only device, the device with C_60_/LiF double CBLs has a lower IPCE at the short wavelengths due to the parasitic absorption in the C_60_ film, and shows a higher IPCE at long wavelengths, owing to the optical spacer effect as well as the contribution of P3HT/C_60_ subcell.

From Table 2, it is noticed that the C_60_ (25 nm)/LiF (8 nm)-based device exhibits a low PCE of 1.10% although this efficiency is still much higher than that (0.06%) of the LiF (8 nm)-only device. The low PCE is the result of the small *J*_sc_ and FF, which is caused by the large *R*_s_. As discussed above, the C_60_ (35 nm)/LiF (5 nm) film has good electrical conductivity due to the formation of the mixed morphology between C_60_ and LiF layers (see Fig. 2). To find the reason for the high resistance of the C_60_ (25 nm)/LiF (8 nm) film, AFM measurements were performed on P3HT:PCBM films without and with the C_60_ (25 nm), LiF (8 nm), and C_60_ (25 nm)/LiF (8 nm) layers deposited on top. As shown in Additional file 1: Figure S3, large spherical aggregates are formed in the C_60_ (25 nm) film while relatively small aggregates are formed in the LiF (8 nm) film, which is similar to the observation in Fig. 2. When depositing 8-nm-thick LiF on top of the C_60_ (25 nm) layer, the morphology (small aggregates) is very similar to that of the pristine LiF film, indicating that the underlying C_60_ aggregates are completely covered by 8-nm-thick LiF. Therefore, we speculate that a thick LiF accumulates at the top of the C_60_ (25 nm)/LiF (8 nm) bilayer film, which hinders the electron extraction and therefore leads to the high *R*_s_ of the device.

## Conclusions

In summary, we have demonstrated that a thick LiF can be used as CBL in P3HT:PCBM-based PSCs by simply introducing a C_60_ layer between the active layer and the LiF layer. The devices with the C_60_/LiF (5 nm) double CBLs exhibit a peak efficiency of 3.65%, while the LiF (5 nm)-only device shows a two times lower PCE of 1.79%. The improved device performance mainly results from the high FF due to the good electrical conductivity of the C_60_/LiF bilayer. In addition, the *J*_sc_ is also improved after introducing a C_60_ interlayer, which can be attributed to the contribution of P3HT/C_60_ subcell as well as the optical spacer effect of C_60_. Further increasing the LiF thickness to 8 nm leads to the rapid decrease of PCE to 1.10 and 0.06% for the C_60_/LiF-based device and LiF-only device, respectively. The decline in PCE of the device with C_60_/LiF (8 nm) double CBLs is caused by the impeded electron transport, owing to the accumulated LiF at the top of the C_60_ (25 nm)/LiF (8 nm) bilayer. All in all, these results indicate that the C_60_/LiF bilayer is a more promising CBL as compared to LiF single layer for fabricating highly efficient and large-scale PSCs.

## Additional file

Additional file 1: Supporting information. **Table S1.** Average photovoltaic performance parameters for the P3HT:PCBM-based PSCs with and without different thicknesses of C_60_ inserted between the active layer and 5-nm-thick LiF layer. **Table S2.** Average photovoltaic performance parameters for the P3HT:PCBM-based PSCs using LiF single and C_60_ (25 nm)/LiF double CBLs with different thicknesses of LiF. **Figure S1.** Photo-CELIV curves for the devices with (a) the LiF (6 nm) single and (b) C_60_ (25 nm)/LiF (6 nm) double CBLs. **Table S3.** Photovoltaic parameters of the P3HT/C_60_ (25 nm)-based PSCs with the P3HT thickness varied from 5 to 100 nm. **Figure S2.** (a) Simulated electric field intensity within the active layer versus the thickness of C_60_ interlayer for the PSCs having the following structure: ITO (150 nm)/PEDOT:PSS (45 nm)/P3HT:PCBM (180 nm)/C_60_ (*x* nm)/Al (120 nm). (b) Absorption spectra of the pristine C_60_ film and the P3HT:PCBM blend films with and without different CBLs deposited on top. (c) Incident photon-to-current conversion efficiency (IPCE) spectra for the devices with and without the C_60_ interlayer. **Figure S3.** AFM height (top) and phase (bottom) images of C_60_ (25 nm), LiF (8 nm), and C_60_ (25 nm)/LiF (8 nm) layers deposited on P3HT:PCBM blend films. (DOC 1663 kb)
