# Supplementary material for: 5-nm LiF as an Efficient Cathode Buffer Layer in Polymer Solar Cells Through Simply Introducing a C60 Interlayer
Source: Nanoscale Res Lett. 2017 Sep 21;12:543. doi: 10.1186/s11671-017-2299-y (PMC5608655; doi:10.1186/s11671-017-2299-y)
Supplement: Additional file 1: — Supporting information. Table S1. Average photovoltaic performance parameters for the P3HT:PCBM-based PSCs with and without different thicknesses of C60 inserted between the active layer and 5-nm-thick LiF layer. Table S2. Average photovoltaic performance parameters for the P3HT:PCBM-based PSCs using LiF single and C60 (25 nm)/LiF double CBLs with different thicknesses of LiF. Figure S1. Photo-CELIV curves for the devices with (a) the LiF (6 nm) single and (b) C60 (25 nm)/LiF (6 nm) double CBLs. Table S3. Photovoltaic parameters of the P3HT/C60 (25 nm)-based PSCs with the P3HT thickness varied from 5 to 100 nm. Figure S2. (a) Simulated electric field intensity within the active layer versus the thickness of C60 interlayer for the PSCs having the following structure: ITO (150 nm)/PEDOT:PSS (45 nm)/P3HT:PCBM (180 nm)/C60 (x nm)/Al (120 nm). (b) Absorption spectra of the pristine C60 film and the P3HT:PCBM blend films with and without different CBLs deposited on top. (c) Incident photon-to-current conversion efficiency (IPCE) spectra for the devices with and without the C60 interlayer. Figure S3. AFM height (top) and phase (bottom) images of C60 (25 nm), LiF (8 nm), and C60 (25 nm)/LiF (8 nm) layers deposited on P3HT:PCBM blend films. (DOC 1663 kb) [file 11671_2017_2299_MOESM1_ESM.doc]

**Supporting Information**

**5-nm LiF as an efficient cathode buffer layer in polymer solar cells through simply introducing a C_60_ interlayer**

Xiaodong Liu^1,2^, L. Jay Guo^3*^ and Yonghao Zheng^1,2*^

^1^*School of Optoelectronic Information, University of Electronic Science and Technology of China (UESTC), Chengdu 610054, PR China*

^2^*Center for Applied Chemistry, University of Electronic Science and Technology of China (UESTC), Chengdu 610054, PR China*

^3^*Department of Electrical Engineering and Computer Science, The University of Michigan, 1301 Beal Ave., Ann Arbor, MI 48109, USA*

* Corresponding author.

*E-mail addresses:* [zhengyonghao@uestc.edu.cn](mailto:zhengyonghao@uestc.edu.cn) (Yonghao Zheng),

[guo@eecs.umich.edu](mailto:guo@eecs.umich.edu) (L. Jay Guo).

**Table S1** Average photovoltaic performance parameters for the P3HT:PCBM based PSCs with and without different thicknesses of C_60_ inserted between the active layer and 5 nm-thick LiF layer

| CBL | *J*_sc_^a)^ (mA/cm^2^) | *V*_oc_^a)^ (V) | FF^a)^ (%) | PCE^a)^ (%) |
| --- | --- | --- | --- | --- |
| LiF | 8.83 ± 0.33 | 0.60 | 33.3 ± 1.0 | 1.76 ± 0.07 |
| C_60_ (3 nm)/LiF | 9.10 ± 0.24 | 0.58 | 55.3 ± 1.2 | 2.91 ± 0.12 |
| C_60_ (5 nm)/LiF | 8.42 ± 0.26 | 0.57 | 66.6 ± 1.6 | 3.19 ± 0.08 |
| C_60_ (8 nm)/LiF | 8.83 ± 0.32 | 0.57 | 67.1 ± 0.8 | 3.38 ± 0.09 |
| C_60_ (12 nm)/LiF | 9.10 ± 0.28 | 0.58 | 65.8 ± 0.7 | 3.46 ± 0.06 |
| C_60_ (15 nm)/LiF | 9.57 ± 0.11 | 0.6 | 62.8 ± 1.5 | 3.58 ± 0.08 |
| C_60_ (25 nm)/LiF | 9.88 ± 0.24 | 0.56 ± 0.01 | 65.4 ± 1.2 | 3.59 ± 0.11 |
| C_60_ (35 nm)/LiF | 8.63 ± 0.24 | 0.55 | 62.3 ± 0.4 | 2.97 ± 0.10 |

^a)^ Average values with standard deviation for five devices processed in the batch.

**Table S2** Average photovoltaic performance parameters for the P3HT:PCBM based PSCs using LiF single and C_60_ (25 nm)/LiF double CBLs with different thicknesses of LiF

| CBL | *J*_sc_^a)^ (mA/cm^2^) | *V*_oc_^a)^ (V) | FF^a)^ (%) | PCE^a)^ (%) |
| --- | --- | --- | --- | --- |
| LiF (0.5 nm) | 9.18 ± 0.12 | 0.59 | 53.3 ± 0.9 | 2.89 ± 0.04 |
| LiF (1 nm) | 9.08 ± 0.21 | 0.57 | 57.5 ± 1.5 | 2.99 ± 0.05 |
| LiF (6 nm) | 7.35 ± 0.14 | 0.58 | 18.3 ± 0.8 | 0.78 ± 0.04 |
| LiF (8 nm) | 1.01 ± 0.05 | 0.58 ± 0.01 | 10.2 ± 0.4 | 0.06 |
| C_60_/LiF (0.5 nm) | 10.16 ± 0.50 | 0.59 | 60.0 ± 0.5 | 3.59 ± 0.19 |
| C_60_/LiF (1 nm) | 9.87 ± 0.16 | 0.56 ± 0.01 | 66.9 ± 0.5 | 3.67 ± 0.09 |
| C_60_/LiF (6 nm) | 8.34 ± 0.23 | 0.54 | 58.6 ± 1.0 | 2.63 ± 0.07 |
| C_60_/LiF (8 nm) | 6.66 ± 0.46 | 0.54 ± 0.01 | 27.2 ± 2.2 | 0.99 ± 0.12 |

^a)^ Average values with standard deviation for five devices processed in the batch.

(a)

(b)

**Fig. S1** Photo-CELIV curves for the devices with (a) the LiF (6 nm) single and (b) C_60_ (25 nm)/LiF (6 nm) double CBLs

**Table S3** Photovoltaic parameters of the P3HT/C_60_ (25 nm) based PSCs with the P3HT thickness varied from 5 to 100 nm

| P3HT thickness | *J*_sc_ (mA/cm^2^) | *V*_oc_ (V) | FF (%) | PCE (%) | *R*_s_ ($\Omega$ cm^2^) | *R*_sh_ ($\Omega$ cm^2^) |
| --- | --- | --- | --- | --- | --- | --- |
| 100 nm | 0.26 | 0.20 | 37.3 | 0.019 | 242.51 | 1644.14 |
| 50 nm | 0.28 | 0.23 | 36.3 | 0.023 | 379.46 | 1672.16 |
| 30 nm | 0.57 | 0.24 | 37.8 | 0.052 | 167.48 | 818.01 |
| 15 nm | 1.12 | 0.26 | 39.1 | 0.114 | 78.55 | 507.27 |
| 10 nm | 1.34 | 0.24 | 41.8 | 0.134 | 48.99 | 470.46 |
| 5 nm | 1.17 | 0.21 | 42.5 | 0.104 | 48.72 | 512.69 |

(a)

(b)

(c)

**Fig. S2** (a) Simulated electric field intensity within the active layer versus the thickness of C_60_ interlayer for the polymer solar cells having the following structure: ITO (150 nm)/PEDOT:PSS (45 nm)/P3HT:PCBM (180 nm)/C_60_ (*x* nm)/Al (120 nm). The complex refractive index of each layer was measured using multiangle ellipsometer or taken from the literature value. The LiF layer is omitted because it does not influence the electric field distribution in polymer solar cells. (b) Absorption spectra of the pristine C_60_ film, and the P3HT:PCBM blend films with and without different CBLs deposited on top. (c) Incident photon-to-current conversion efficiency (IPCE) spectra for the devices with and without the C_60_ interlayer


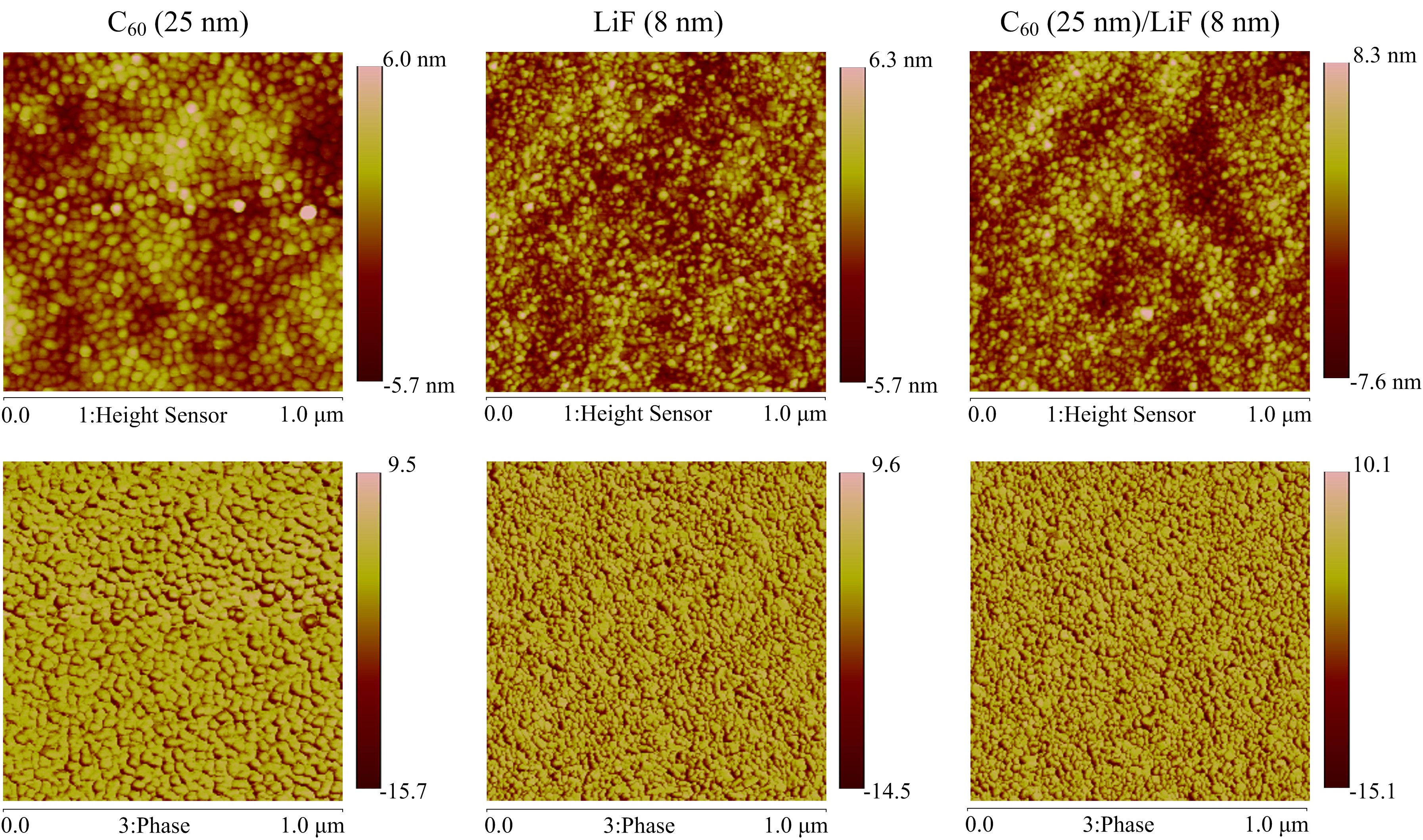


**Fig. S3** AFM height (top) and phase (bottom) images of C_60_ (25 nm), LiF (8 nm) and C_60_ (25 nm)/LiF (8 nm) layers deposited on P3HT:PCBM blend films. The corresponding rms roughnesses are 1.69, 1.73 and 2.27 nm, respectively
